# Trueness and precision of 5 intraoral scanners in the impressions of single and multiple implants: a comparative in vitro study

**DOI:** 10.1186/s12903-019-0792-7

**Published:** 2019-06-06

**Authors:** Francesco Guido Mangano, Uli Hauschild, Giovanni Veronesi, Mario Imburgia, Carlo Mangano, Oleg Admakin

**Affiliations:** 1 Department of Prevention and Communal Dentistry, Sechenov First Moscow State Medical University, Moscow, Russia; 2 Department of Post-graduate Education, Faculty of Oral and Dental Medicine, J.W. Goethe University, Frankfurt, Germany; 3 Department of Medicine and Surgery, Research Center in Epidemiology and Preventive Medicine, University of Varese, Varese, Italy; 4 Private Practice, Palermo, Italy; 5 Department of Dental Sciences, Vita and Salute University San Raffaele, Milan, Italy; 6 Department of Prevention and Communal Dentistry, Sechenov First Moscow State Medical University, Moscow, Russia

**Keywords:** Intraoral scanners, Oral implantology, Trueness, Precision

## Abstract

**Background:**

Until now, a few studies have addressed the accuracy of intraoral scanners (IOSs) in implantology. Hence, the aim of this in vitro study was to assess the accuracy of 5 different IOSs in the impressions of single and multiple implants, and to compare them.

**Methods:**

Plaster models were prepared, representative of a partially edentulous maxilla (PEM) to be restored with a single crown (SC) and a partial prosthesis (PP), and a totally edentulous maxilla (TEM) to be restored with a full-arch (FA). These models were scanned with a desktop scanner, to capture reference models (RMs), and with 5 IOSs (CS 3600®, Trios3®, Omnicam®, DWIO®, Emerald®); 10 scans were taken for each model, using each IOS. All IOS datasets were loaded into a reverse-engineering software where they were superimposed on the corresponding RMs, to evaluate trueness, and superimposed on each other within groups, to determine precision. A statistical analysis was performed.

**Results:**

In the SC, CS 3600® had the best trueness (15.2 ± 0.8 μm), followed by Trios3® (22.3 ± 0.5 μm), DWIO® (27.8 ± 3.2 μm), Omnicam® (28.4 ± 4.5 μm), Emerald® (43.1 ± 11.5 μm). In the PP, CS 3600® had the best trueness (23 ± 1.1 μm), followed by Trios3® (28.5 ± 0.5 μm), Omnicam® (38.1 ± 8.8 μm), Emerald® (49.3 ± 5.5 μm), DWIO® (49.8 ± 5 μm). In the FA, CS 3600® had the best trueness (44.9 ± 8.9 μm), followed by Trios3® (46.3 ± 4.9 μm), Emerald® (66.3 ± 5.6 μm), Omnicam® (70.4 ± 11.9 μm), DWIO® (92.1 ± 24.1 μm). Significant differences were found between the IOSs; a significant difference in trueness was found between the contexts (SC vs. PP vs. FA). In the SC, CS 3600® had the best precision (11.3 ± 1.1 μm), followed by Trios3® (15.2 ± 0.8 μm), DWIO® (27.1 ± 10.7 μm), Omnicam® (30.6 ± 3.3 μm), Emerald® (32.8 ± 10.7 μm). In the PP, CS 3600® had the best precision (17 ± 2.3 μm), followed by Trios3® (21 ± 1.9 μm), Emerald® (29.9 ± 8.9 μm), DWIO® (34.8 ± 10.8 μm), Omnicam® (43.2 ± 9.4 μm). In the FA, Trios3® had the best precision (35.6 ± 3.4 μm), followed by CS 3600® (35.7 ± 4.3 μm), Emerald® (61.5 ± 18.1 μm), Omnicam® (89.3 ± 14 μm), DWIO® (111 ± 24.8 μm). Significant differences were found between the IOSs; a significant difference in precision was found between the contexts (SC vs. PP vs. FA).

**Conclusions:**

The IOSs showed significant differences between them, both in trueness and in precision. The mathematical error increased in the transition from SC to PP up to FA, both in trueness than in precision.

## Background

Intraoral scanners (IOSs) are powerful devices for acquiring an optical impression of dental arches, able to replace the conventional techniques with trays and materials (alginate, polyvinylsiloxane, polyether) that have always been unwelcome to patients [1–3]. IOSs, for this reason and for their different possible applications—diagnosis and acquisition of study models [4], fixed prostheses [2, 3], guided implant surgery [5], orthodontics [6]—are spreading in the dental world and an increasing number of dentists purchase such machines and adopt this technology [1–3, 6, 7]. IOSs project a light source (generally a structured light grid with a known geometry; or a laser beam) on the surface of the teeth and capture its deformation with powerful cameras; this data is reworked by the acquisition software that generates a point cloud, which is then triangulated to produce a mesh [1–3]. This mesh represents the direct reconstruction of the surface of the object [1–3]. With IOSs, the dentate models are directly captured; there is no need to pour a plaster cast from a negative impression, as with the conventional alginate, polyvinylsiloxane, or polyether impressions. This is theoretically an advantage, because all the possible errors related to the transition from negative to positive are eliminated; also, the virtual model can be quickly emailed to the dental laboratory, at no cost [1–3, 6, 7].

Even though the clinicians often focus their attention on speed and ease of use, as well as on practical features such as the absence of powder, the color, and the possibility of exporting files without having to pay any release fee, it must be noted that the mathematical quality of the files derived from the IOS is more important [1]. The main mathematical features an IOS should possess are accuracy [1, 7–11] and resolution [12].

Accuracy is key in all clinical applications in prosthesis, whether with natural teeth or with implants—an IOS should be able to detect an accurate impression [8–11]. In metrics and engineering, accuracy is defined as the “closeness of agreement between a measured quantity value and a true quantity value of a measurand” (JCGM 200:2012; ISO 5725–1, 1994). Ultimately, accuracy is the sum of trueness and precision [8–11]. Trueness, usually expressed in terms of bias, is the “closeness of agreement between the expectation of a test result or a measurement result and a true value” [9, 10]. Precision is defined as the “closeness of agreement between indications or measured quantity values obtained by replicate measurements on the same objects under specified conditions” [9, 10]. In other words, the ideal IOS should be able to reconstruct and therefore reproduce as faithfully as possible the surface of the scanned object, i.e., it should possess high trueness; and it should have high precision, giving consistent and repeatable results without any deviations when scanning the same object [10, 11].

It is rather simple to measure, in vivo, the precision of an IOS: it is sufficient to capture different scans of the same arch, one after the other, save these 3D models, and, via reverse-engineering software, overlap them. In this context, minimal deviations between the models indicate high precision of the IOS. Calculating the trueness in vivo instead is more difficult; in order to do it, via reverse engineering software, we need in fact a reference model (RM), onto which we can superimpose our intraoral scans [9, 10]. To date, a RM can be captured only by means of sophisticated machines such as articulated arms or coordinate measuring machines (CMMs), i.e., devices that physically probe the surface of the object for detailed 3D information; alternatively, powerful industrial or desktop optical scanners can be used for this purpose [10]. Since it is not possible to detach the patient’s dental arches and place them inside a CMM or an industrial optical scanner to get a RM, it is impossible to calculate the trueness of an IOS in vivo.

Finally, in IOS, the resolution is given by the density of the point cloud and therefore by the number of triangles that constitutes the mesh [12]. This resolution is essential for the visualization of details such as the margin or preparation line of a natural tooth [12], but it is of lesser importance in the case of implants, where the impression captures only a position and the scanbody is then replaced by pre-formed components from a library, on which the computer assisted design (CAD) modeling takes place [13, 14]. Therefore, there are important differences between scanning of natural teeth and scanning of implants, and the latter could be defined as easier.

However, only a few clinical studies have been published so far in the literature on the full-digital workflow, starting from intraoral scanning, for implant-supported rehabilitations [1–3, 7, 13–17]. Most of these studies reported good results with single implants [3, 7, 13–17], while few have focused on the restoration of multiple implants [18, 19]. It seems that the IOSs have difficulty in capturing, in vivo, accurate impressions for the design and manufacture of long-span restorations [20, 21]. To date, in particular, the scientific literature does not support the use of IOSs for impression capture on multiple implants, aimed at the manufacture of extended implant-supported restorations as full arches (FAs) [20, 21]. This limitation is determined by the acquisition methods of IOS and therefore the difficulty of reconstructing extended surfaces [22].

Since the IOSs that are currently on the market have different characteristics (acquisition methods and reconstruction algorithms) and today few studies have addressed their accuracy [12, 23–28], particularly in implantology [9–11, 26–28], the aim of the present in vitro study was to assess the trueness and precision of 5 different IOSs in the impressions of single and multiple implants, and to compare them.

## Methods

### Study casts

**Fig. 1:**
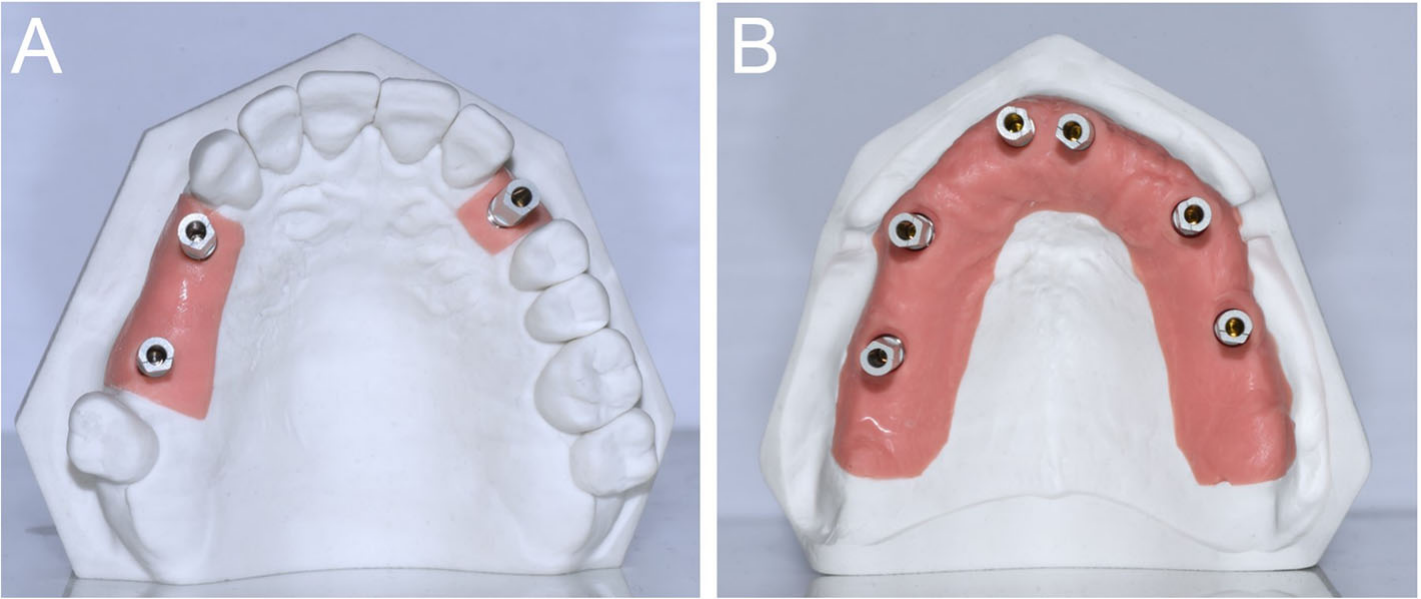
Two different plaster models were prepared, representing three different situations in the maxilla. The first model (**a**) was a partially edentulous maxilla (PEM), with an implant analog in position #23 (left upper canine), to simulate the situation of an implant-supported single crown (SC), and with two implant analogs in position #14 and #16 (respectively right first premolar and first molar), to simulate the situation of an implant-supported partial prosthesis (PP). The second model (**b**) was a totally edentulous maxilla (TEM), with implant analogs in position #11, #14, #16, #21, #24 and #26 (right and left central incisors, first premolars and first molars), to simulate a situation of an implant-supported fixed full-arch (FA) prosthesis. All models presented pink gums in the areas of implant analogs, with high-precision non-reflective polyether-ether-ketone (PEEK) scanbodies (Megagen®, Daegu, South Korea) screwed on the implant analogs

The dental laboratory prepared two different plaster models, representing three different situations/contexts in the maxilla. The first model was a partially edentulous maxilla (PEM), with an implant analog in position #23 (left upper canine) to simulate the situation of an implant-supported single crown (SC), and with two implant analogs in position #14 and #16 (respectively right first premolar and first molar) to simulate the situation of an implant-supported partial prosthesis (PP) (Fig. 1a). The second model was instead a totally edentulous maxilla (TEM), with implant analogs in position #11, #14, #16, #21, #24, and #26 (right and left central incisors, first premolars and first molars), to simulate the situation of an implant-supported fixed FA prosthesis (Fig. 1b). All models presented pink gums in the areas of implant analogs. High-precision non-reflective polyether-ether-ketone (PEEK) scanbodies (Megagen®, Daegu, South Korea) were screwed on the implant analogs; PEEK was selected because it does not reflect light and therefore facilitates acquisition with three-dimensional (3D) scanners [29].

### Design of the study

The present in vitro study compared 5 different IOSs that are currently available on the market (CS 3600®, Carestream Dental, Atlanta, Georgia USA; Trios3®, 3Shape, Copenhagen, Denmark; CEREC Omnicam®, Dentsply-Sirona, York, Pennsylvania, USA; DWIO®, Dentalwings, Montreal, Quebec, Canada; and Emerald®, Planmeca, Helsinki, Finland), with the aim of investigating their trueness and precision, and therefore their accuracy, within oral implantology.

The design of the study was the following: the two models with the scanbodies in position were acquired with a desktop scanner of industrial derivation (Freedom UHD®, Dof Inc., Seogdong-gu, Seoul), and three scans were captured for each of the models. These scans were subsequently imported and cut into a reverse-engineering software (Geomagic Studio 2012®, Geomagic, Morrisville, North Carolina, USA), using a preconfigured cutting tool (in order to always reproduce the same cuts). The resulting three preconfigured cuts corresponded respectively to: (1) the single implant (to be restored with a SC) in conjunction with the two adjacent teeth; (2) the two implants (to be restored with a PP) in conjunction with their two adjacent teeth; and (3) the six implants (to be restored with a fixed FA). These surface meshes (nine in all, three per type) were saved as standard triangulation language (.STL) files, and overlapped each other, within each group (single on single, partial on partial, total on total) inside the reverse-engineering software. These superimpositions were performed to validate the reference tool, evaluating the deviations between the different files acquired, and thus to select the virtual RM, one by type, to be used later as a basis for the overlap of the various IOS files (trueness evaluation).

Once the reference tool was validated and the three RMs were selected, a single operator expert in digital dentistry began to scan the plaster models with each of the IOSs available. In all, 10 scans were captured for each of the three situations (SC, PP, FA) with each of the IOSs. In the case of the PEM, therefore, the operator did not perform a complete scan of the model, but only captured the area of the pink gingiva, of the scanbody, and of the adjacent teeth (single implant); and the area of the pink gingiva, the two scanbodies, and the adjacent teeth (two implants). In the case of the TEM, the operator captured the whole area of the pink gingiva and the scanbodies (six implants). To avoid the effects of operator fatigue, the sequence of scans was randomized and the scans were captured sequentially, one after the other, with the different machines, at intervals of 5 min from each other. In all cases, and for all IOSs, the operator used a zig-zag technique: he started from the buccal side, carried occlusal and then palatal, and then returned to the occlusal, progressing constantly. The movement described by the tip of the scanner was therefore an arc, moving slowly to fly over the teeth and scanbodies, capturing all details possible but only in the area of interest. All IOSs were used under the same environmental conditions—in a room with a temperature of 22C° (humidity at 45%, air pressure around 750 ± 5 mm).

### The scanners

The main characteristics of all IOSs were summarized in Table 1. A reference scanner (Freedom UHD®, Dof Inc., Seogdong-gu, Seoul, Korea) of industrial derivation was used for the acquisition of the RMs in this study. Freedom UHD uses structured light (white LED light) and acquires thanks to two 5.0 MegaPixel cameras, using the patented stable scan stage (SSS) technology. The SSS system allows the cameras to move above and around the model to be scanned. The cameras and lights rotate around the center of the scan plate, while the model remains stationary; this allows one to capture all the details of the model effectively and quickly (in less than 50 s). The scanner has a certified accuracy of 5 μm and generates. STL files immediately usable by any CAD. The scanner weighs 15 kg, has dimensions of 330 × 495 × 430 mm, is powered at 110–240 V, 50–60 Hz, and works with Windows operating systems 7, 8, and 10 (64-bit).

**Table 1:** The five intraoral scanners used in this study

	Producer	Technology of acquisition	Powder	Colour	System
CS 3600®	Carestream Dental, Atlanta, Georgia, USA	Structured light-Active Speed 3D Video™	No	Yes	Proprietary files (.CSZ), but also open formats (.PLY,.STL) immediately available
Trios3®	3-Shape, Copenhagen, Denmark	Structured light –Confocal microscopy and Ultrafast Optical Scanning™	No	Yes	Proprietary files (.DCM) available, but possibility to export .STL files via the new Trios on Dental Desktop®
Omnicam®	Dentsply-Sirona, York, Pennsylvania, USA	Structured light -Optical triangulation and confocal microscopy	No	Yes	Proprietary files (.CS3,.SDT,.CDT,.IDT) are available, but possibility to export .STL files via the Cerec Connect®
DWIO®	Dentalwings, Montreal, Quebec, Canada	Blue laser-Multiscan Imaging™ technology	No	No	Proprietary files (.XORDER), but also open Formats (.STL) immediately available
Emerald®	Planmeca, Helsinki, Finland	Red, green and blue lasers-Projected Pattern Triangulation™	No	Yes	Open formats (.PLY,.STL) immediately available

CS 3600®, launched in 2016, is a structured LED light scanner. CS 3600® is fast thanks to the Intelligent Matching System™, which allows the software to connect the scanned images very quickly and build the mesh continuously, without interruption. CS 3600® is equipped with interchangeable and autoclavable tips, of different sizes and with different orientations, to facilitate scanning even in the most difficult areas. The IOS easily connects to the computer through a USB port, does not require the use of powder, and is able to provide HD full-color images in 3D, which are a valuable marketing tool from the patient’s perspective and at the same time help the clinician in identifying the margin line (when used in scanning on natural teeth). Finally, CS 3600® is an open IOS, which produces proprietary files (.CSZ) with color information, which can be opened in the simplified Carestream CAD (CS Restore®) for design and the subsequent manufacture of a whole series of simple restorations (inlays, onlays, veneers, single crowns), but also open files (.PLY,. STL) that can be processed by any dental CAD. One of these formats in particular (.PLY), although usable by any CAD, allows one to keep the color information. CS 3600® does not require the payment of any annual or monthly fee for use or for the unlocking of proprietary files. There are no restrictions for laboratories in the use of color (.PLY) or monochromatic (.STL) files of CS 3600®. The IOS is suitable for the acquisition of images for the design of a wide range of prosthetic restorations (inlays, onlays, veneers, single crowns, and bridges up to bars) and for the acquisition of the dento-gingival information to be combined with the bone, obtained with the cone-beam computed tomographies (CBCTs) produced by Carestream (CS 9300®, CS 8100®, and others) in the workflow in guided surgery. Finally, CS 3600® is used for the diagnosis and design of orthodontic devices. In the present study, the release V3.0 (09–2017) of the acquisition software was used.

Trios3® has been released by the 3Shape Company in 2015. Available in different versions (trolley with touch screen, built-in version in dental unit, and version connected to a laptop via USB) with a straight pen-grip handle or with a pistol-shaped handle (320 × 56 × 16 mm); since 2017 it implements a wireless version, in which the scanner is connected to a laptop via WiFi, eliminating the need for connection cables. Trios3® is a structured light scanner that uses confocal microscopy and Ultrafast Optical Scanning™ technology to capture more than 3000 two-dimensional images per second. It then combines up to 1000 3D digital pictures. It is powder-free and produces high-quality color images implementing Real Color Scan™, HD Photo Function™, and Digital Shade Determination™ technologies. With Trios3®, the colour scanning can help to differentiate the natural tooth structure and the gingival tissues, and therefore it may help dentists to identify the margin lines; in addition, it represents a valuable marketing tool with patients. Trios3® has a big wand, but this is not a limitation because this tip can be used to avoid scanning of unwanted tissues (tongue, cheeks, lips). Trios3® is still considered to be a closed system; in fact, it generates proprietary files (.DCM) which can be opened by the 3Shape CAD software (3Shape Dental System®), one of the most widespread design platforms available on the market, via the proprietary cloud-based platform (Trios Inbox®) or setting up a direct connection via Direct Connect®, through which data are fed into the dental system and read out from there. However, in the present study, the software version 1.6.4 (Trios on Dental Desktop®) has been used. Trios on Dental Desktop® is the new 3Shape unified platform that integrates all digital workflows into an intuitive user interface, with integrated HD intraoral camera, patient monitoring, smile design, treatment simulator, shade measurement, and, for the first time,. STL scan export. The CAD software from 3Shape allows design of all kinds of prosthetic restorations and frameworks (inlays, onlays, veneers, crowns, bridges, bars); in addition, modules for implant (3Shape Implant Studio®) and orthodontic planning (3Shape Ortho Analyzer®) are available. However, 3Shape still has no dedicated milling machines for in-office, chairside restorations.

CEREC Omnicam® has long been the most sophisticated IOS of the Dentsply-Sirona, at least until the recent presentation, at the annual fair in Dubai in 2019, of the company’s new product, Primescan®. Omnicam® represents the development and technological evolution of the previous IOSs produced by the German Sirona (CEREC Bluecam®, available since 2009, and Apollo DI®), the first company to introduce intraoral scanning in the world, and therefore long monopolising the market. Introduced in 2012 and available in two different versions (trolley, Omnicam AC®, and tabletop, Omnicam AF®) Omnicam® is a structured light scanner that uses a white LED and works under the principle of optical triangulation and confocal microscopy. Extremely fast, it does not require the use of powder and incorporates the color inside the reconstructed 3D model. The scanner is of medium size (228 × 16 × 16 mm), but the tip is not too large and this makes scanning even easier in the posterior areas (maxillary or mandibular third molars). The acquisition software is as powerful as the dedicated CAD, and the workflow can be done directly at the chairside, using the proprietary CAD software or the cloud-based platform (CEREC Connect®). CEREC Omnicam® is theoretically a closed system, because it produces proprietary files (.CS3,. SDT,. CDT,. IDT) that can only be opened by CAD software of the same company; however, with the introduction of CEREC Connect® the system has been partially opened, giving the user the possibility to transform the proprietary files into. STL, which can be used by any other CAD software. In this study, we have used the software CEREC Connect 4.4.4®, and all proprietary files have been converted into. STL via Inlab software (16.0). Sirona has always had cutting-edge chairside solutions, such as the Chairside software 4.4® in combination with the 3 + 1-axis CEREC MC® milling unit (X / XL); however, the company also has powerful laboratory tools such as the inLAB15® CAD software and the MC X5® milling machine. The computer assisted design/ computer assisted manufacturing (CAD/CAM) system by Sirona allows the clinician and the laboratory to design and mill a series of prosthetic restorations and frameworks (inlays, onlays, veneers, crowns, bridges, bars). In addition, Omnicam® has a software for guided surgery (CEREC Guide®), enabling the chairside manufacture of surgical templates, and a software for orthodontic applications (CEREC Ortho®).

DWIO®, presented in its first version during the Chicago Midwinter Meeting of 2015, is a laser scanner that uses a Multiscan Imaging™ technology and integrates five pairs of miniaturized 3D scanners into the tip of the handpiece. The main feature of this IOS is that the handpiece is really thin and light and it has about the same dimensions as a common implant handpiece; it therefore allows one to capture even difficult preparation areas, without effort and without causing any discomfort to the patient. The scanner, which initially required the use of powder, is, in the latest version (used in this study, the version 2.1.0.421) powder-free and as output has proprietary files (.XORDER) and free. STL files that can be open from any CAD and do not require payment of fees for unlocking. The scanner is very fast (< 60 s per arcade) but does not rebuild the object in color. It is available in two versions, both of which feature an innovative voice and gesture control system, to allow the clinicians to control the computer without having to remove their gloves during the scan. The DWIO® is integrated into the powerful CAD system from Dentalwings, one of the best known and used worldwide. DWIO® is indicated for the capture of models for the fabrication of several prosthetic restorations (inlays, onlays, veneers, crowns, bridges) and for the guided surgery as well, thanks to the CoDiagnostiX® software, one of the most important on the market, always developed by Dentalwings.

The latest addition to the Planmeca family, and launched in 2017, Emerald® is a laser scanner (red, green, and blue lasers) that uses Projected Pattern Triangulation™ technology to quickly capture 3D images of dental arches. This IOS reconstructs the models in color and does not require the use of powder. In addition, it is rather small in size (41 × 45 × 249 mm) and light (235 g with the tip mounted) and has autoclavable tips of different sizes to allow the operator to scan even the most difficult areas (posterior sectors, third molars). The scanner easily connects to the computer via USB-3 / USB-C port but can even be integrated into the dental unit, with foot control. The scanner exports free files (.PLY /. STL) that, whether integrating the color information or not, can be opened by the software of the company (Planmeca Romexis® and Planmeca PlanCAD® Easy software suites) as well as freely from any CAD software available on the market. Since Planmeca is a renowned and well-known home for the production of high quality X-ray and CBCT devices (such as ProMax3D®), the Emerald® scanner represents not only the access door for digital prosthetics, with the possibility of designing a whole series of restorations (inlays, onlays, veneers, crowns, bridges, bars), but also the ideal tool to acquire dento-gingival models for guided surgery. 3D models acquired with Emerald® are easily combined with 3D acquisitions of bone volumes using CBCT for planning and making templates for guided implant surgery. In this study we used Planmeca Romexis 5.1.0 software for scanning.

### Trueness and precision

The evaluation of the trueness and precision of the models acquired through the different IOSs studied was as previously reported [9, 10]. In short, all the models acquired with the different IOSs, and their corresponding three RMs, were imported into a reverse-engineering software (Geomagic Studio 2012). The models were then cut/trimmed using dedicated templates through the function “cut with planes” in order to make them uniform. These uniform models were then saved in specific folders and were ready for superimposition. The power of the superimposition algorithms of the reverse-engineering software in use had already been validated in a previous study [9] through the duplication of an identical model, moved in space and then superimposed on itself; these tests had confirmed the absolute reliability of the aforementioned algorithms [9]. For the evaluation of trueness, each of the IOS scans was superimposed onto the corresponding RM, obtained with the desktop scanner. The process basically consisted of three steps. First, a rough alignment was manually performed by means of three fixed points that were identified on the surface of the implant scanbodies in the IOS and RM models. Once this manual phase had been completed, we proceeded to the surface alignment through the “best fit” superposition algorithm of the reverse-engineering software. This algorithm made the final superimposition of the various. STL files derived from IOS on the corresponding RMs. The parameters set for this superimposition were a minimum of 100 iterations per case, for the registration that occurred thanks to a RICP (“robust-iterative-closest-point”) algorithm. The distances between the IOS models and the corresponding RMs were minimized using a point-to-plane method; congruence between specific corresponding structures was calculated. Thanks to these superimposing algorithms, the mean ± standard deviation (SD) of the distances between the two superimposed models was calculated by the software. Finally, the software allowed the generation of a colorimetric map for the immediate visualization, in 3D, of the distances between the models. This was done through the “3D deviation” function and the colorimetric map quantified the distances between specific points, globally and in all space planes. The color maps indicated inward (blue) or outward (red) displacement between overlaid structures, whereas a minimal change was indicated by green color. The same setting of the colorimetric map was set, for all three models (SC, PP, FA); the color scale ranged from a maximum deviation of + 100 and − 100 μm, with the best result given by the deviations between + 30 and − 30 μm (green color). For the precision evaluation, the working method was identical: a first superimposition by points followed the overlap for surfaces and the generation of the colorimetric map. However, IOS-derived models were overlapped on each other, within each group, and not on the corresponding RM (which was not used). The choice of the IOS models to be superimposed was based on a randomized design, which led to a total of 10 overlaps within each group; the precision of each IOS could therefore be obtained, and expressed as a mean (±SD).

### Statistical analysis

A careful statistical analysis was performed, for mean and absolute deviations. Trueness was defined from the superimposition of each scan (10 scans per each IOS group) on the corresponding RM, captured with the desktop scanner. The analysis was first stratified by the context (SC, PP, and FA). For each scanner, the mean trueness and its SD were calculated from analysis of variance, and all possible pairwise comparisons between IOSs were tested, using the Tukey investigation for multiple comparisons. In the footnotes to the tables, the minimum significant mean differences after the Tukey’s correction were reported. Bartlett’s test was used for the assumption of homoscedasticity of variances across groups. The same analyses were replicated for precision, defined from the superimposition between different scans made with the same IOS. For this analysis, 10 comparisons for each scanner were available per each IOS type. Finally, we compared mean trueness and precision of any given scanner, by context (SC vs. PP vs. FA), using separate *t*-tests, with Satterthwaite approximation for the variance. All statistical analyses were conducted using a powerful statistical package (SAS software release 9.4®, SAS Institute, Cary, NC).

## Results

**Fig. 2:**
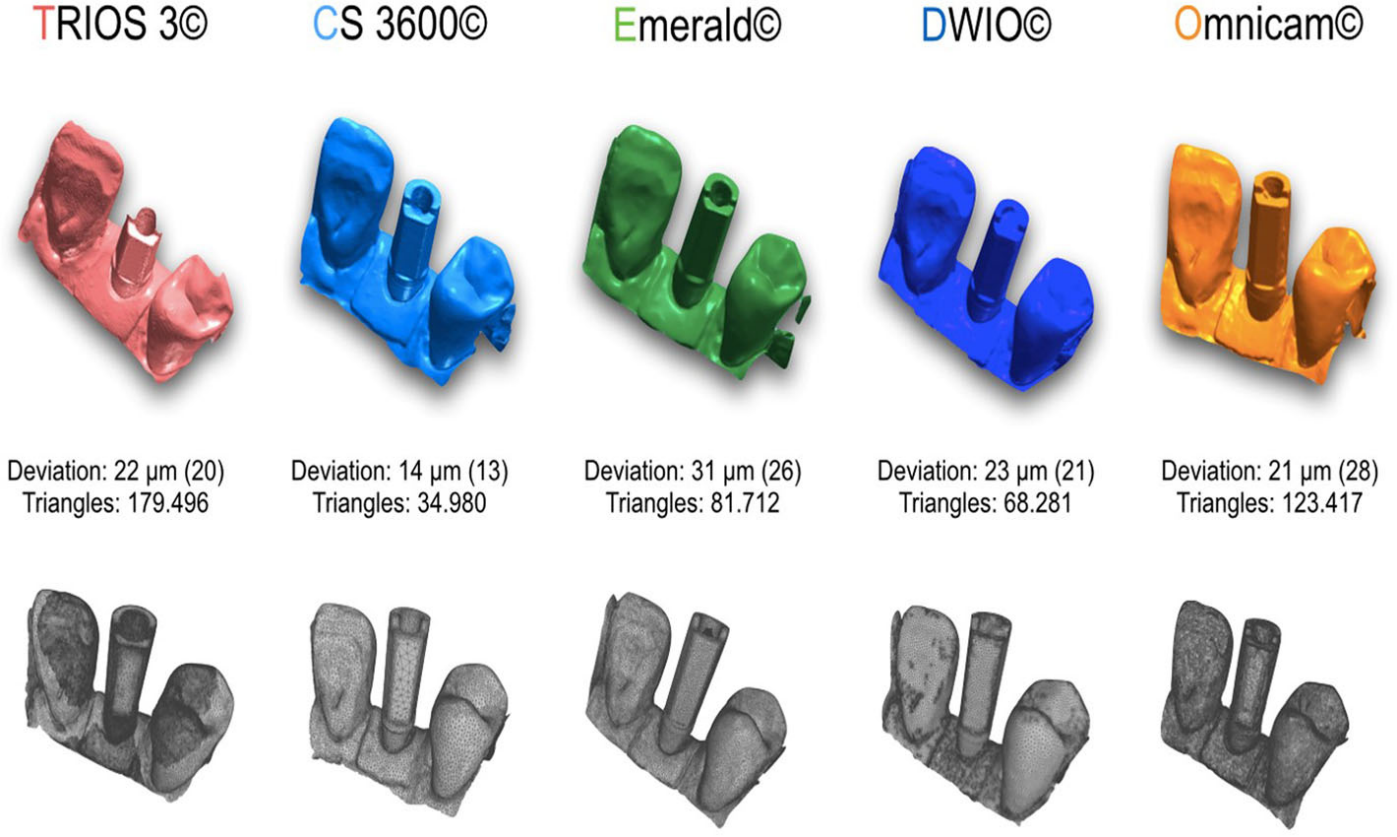
Single crown (SC): best result in trueness (standard deviation), in μm, for the 5 examined scanners, and the number of triangles composing each mesh

**Fig. 3:**
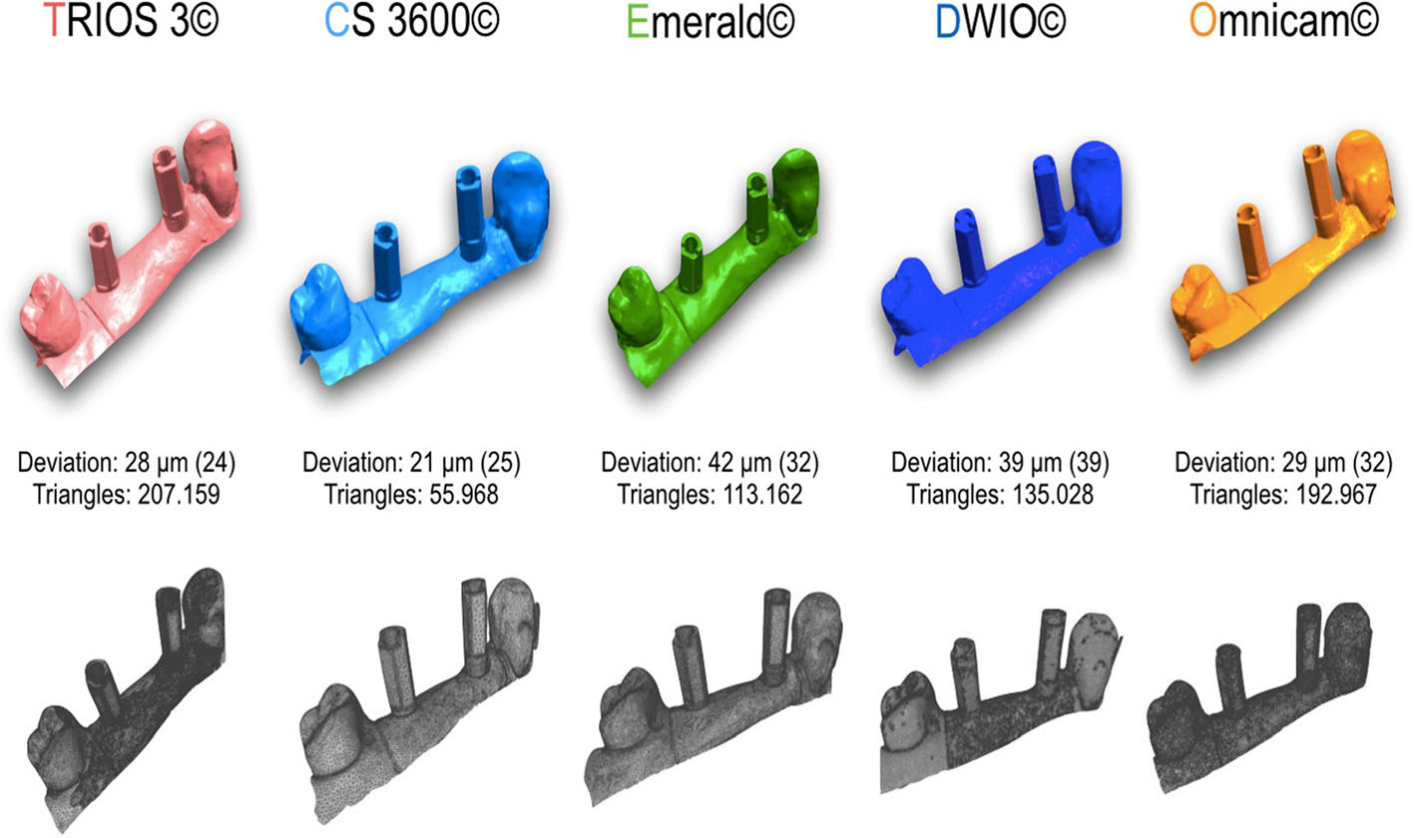
Partial prosthesis (PP): best result in trueness (standard deviation), in μm, for the 5 examined scanners, and the number of triangles composing each mesh

**Fig. 4:**
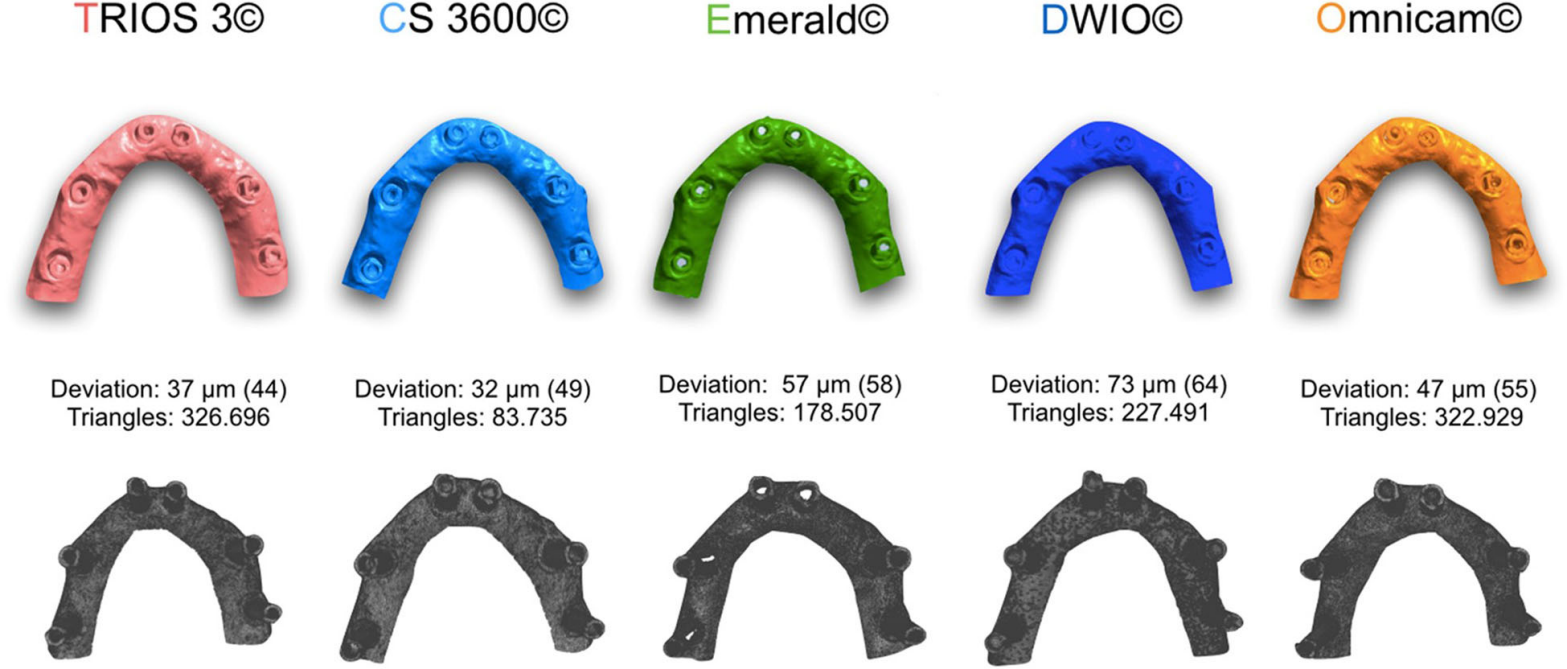
Full arch (FA): best result in trueness (standard deviation), in μm, for the 5 examined scanners, and the number of triangles composing each mesh

**Fig. 5:**
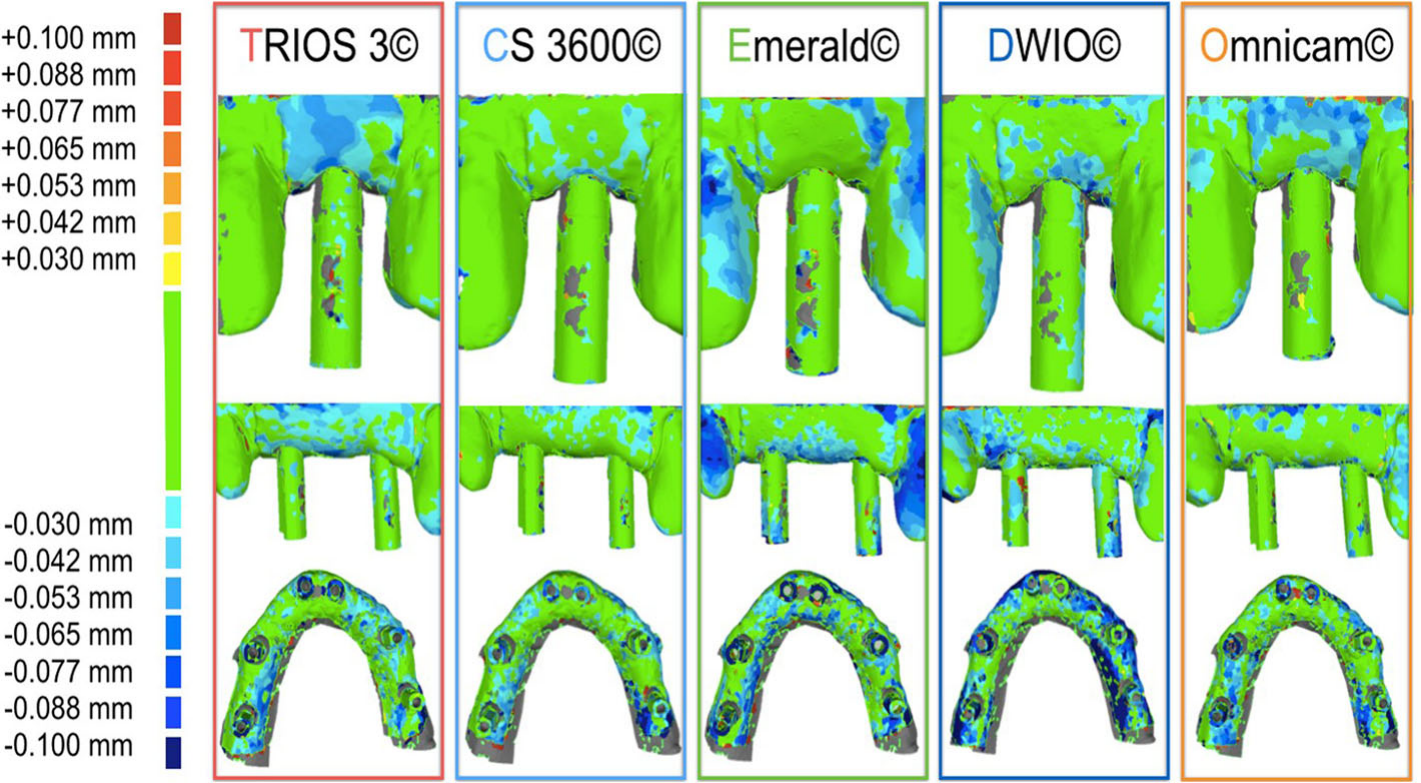
Trueness in the single crown (SC), partial prosthesis (PP) and full-arch (FA) with the 5 examined intraoral scanners (IOSs): colorimetric maps. The color maps indicated inward (blue) or outward (red) displacement between overlaid structures, whereas a minimal change was indicated by a green color. For all three models (SC, PP, FA): the color scale ranged from a maximum deviation of + 100 μm and − 100 μm, with the best result given by the deviations comprised between + 30 μm and − 30 μm (green color)

**Fig. 6:**
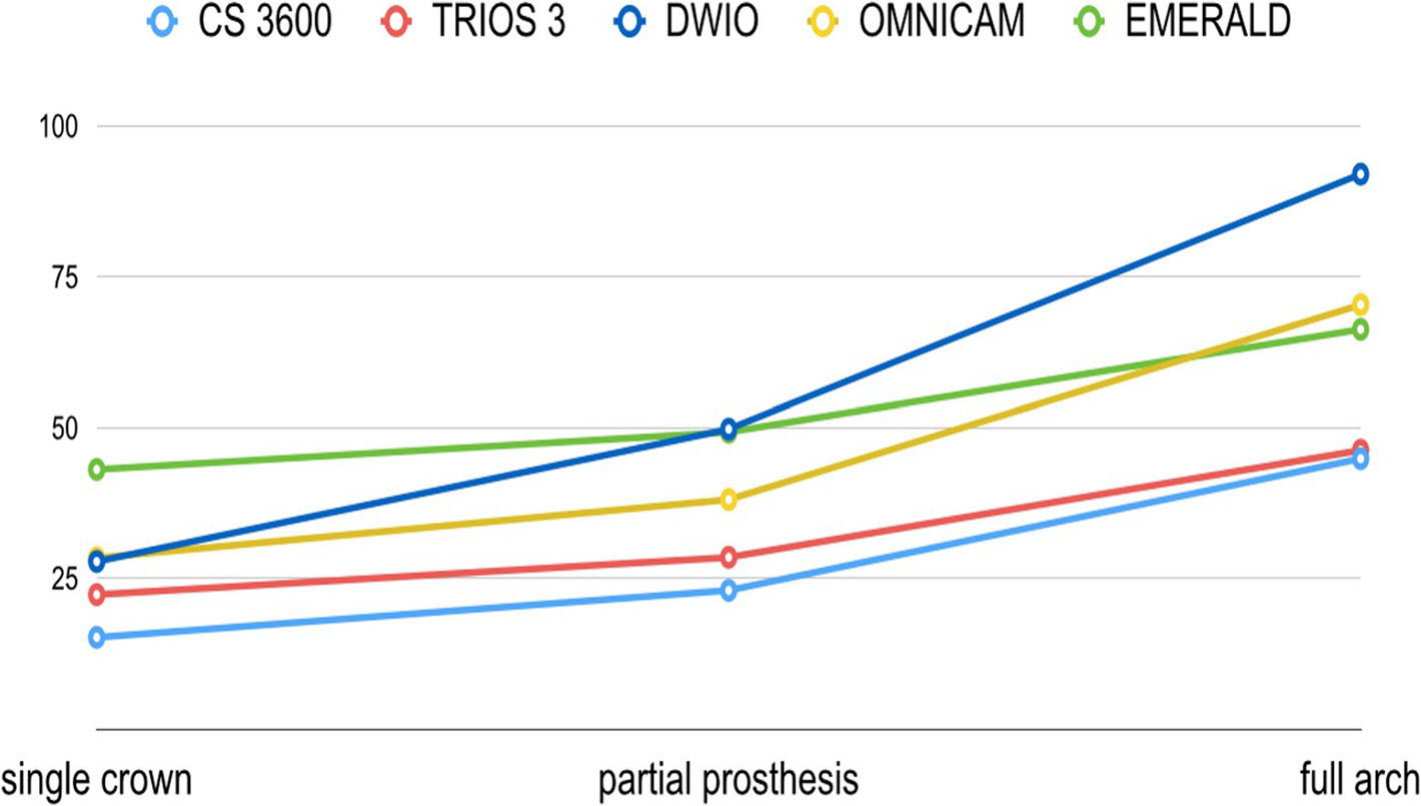
Changes in mean trueness (standard deviation), in μm, for the 5 examined scanners, in the different applications (single crown, SC vs. partial prosthesis, PP vs. full-arch, FA)

The trueness results are summarized in Table 2 and in Figs. 2, 3, 4, 5 and 6. In brief, in the SC, CS 3600® had the best trueness (15.2 ± 0.8 μm), followed by Trios3® (22.3 ± 0.5 μm), DWIO® (27.8 ± 3.2 μm), Omnicam® (28.4 ± 4.5 μm), and Emerald® (43.1 ± 11.5 μm). CS 3600® was statistically truer than DWIO®, Omnicam®, and Emerald®; while Trios3®, DWIO®, and Omnicam® were statistically truer than Emerald®. In the PP, CS 3600® had the best trueness (23 ± 1.1 μm), followed by Trios3® (28.5 ± 0.5 μm), Omnicam® (38.1 ± 8.8 μm), Emerald® (49.3 ± 5.5 μm), and DWIO® (49.8 ± 5.0 μm). CS 3600® and Trios3® were statistically truer than Omnicam®, Emerald®, and DWIO®; while Omnicam® was statistically truer than Emerald® and DWIO®. Finally, in the FA, CS 3600® had the best trueness (44.9 ± 8.9 μm), followed by Trios3® (46.3 ± 4.9 μm), Emerald® (66.3 ± 5.6 μm), Omnicam® (70.4 ± 11.9 μm), and DWIO® (92.1 ± 24.1 μm). CS 3600® and Trios3® were statistically truer than Emerald®, Omnicam®, and DWIO®; while Emerald® and Omnicam® were statistically truer than DWIO®. A statistically significant difference in trueness was found, for each scanner, between the different contexts (SC vs. PP vs. FA).

**Table 2:** Mean trueness and its standard deviation (SD) in micrometers (μm) with single crown (SC), partial prosthesis (PP) and full-arch (FA), and *p* values testing the scanner by context interaction. *N* = 10 scans for each scanner and implant type

Scanner	Single Crown (SC)	Partial prosthesis (PP)	Full arch (FA)	*p*-value^1^
	Mean ± SD	Mean ± SD	Mean ± SD	
Trios 3®	22.3 ± 0.5†	28.5 ± 0.5†,‡,•	46.3 ± 4.9†,‡,•	< 0.0001
CS 3600®	15.2 ± 0.8‡,#,§	23.0 ± 1.1^,§,#	44.9 ± 8.9^,§,#	< 0.0001
Emerald®	43.1 ± 11.5†,‡,•,^	49.3 ± 5.5†,^,°	66.3 ± 5.6†,^,°	< 0.0001
DWIO®	27.8 ± 3.2#,•	49.8 ± 5.0‡,§,*	92.1 ± 24.1‡,§,°,*	< 0.0001
Omnicam®	28.4 ± 4.5§,^	38.1 ± 8.8•,#,°,*	70.4 ± 11.9•,#,*	< 0.0001

The same symbol after SD indicates differences in trueness between scanner pairs (Tukey-adjustment for multiple comparison). Minimum significant difference across scanners: 7.3 μm, 6.6 μm, 16.8 μm for single crown (SC), partial prosthesis (PP) and full arch (FA), respectively. ^1^*p*-value testing the interaction between scanner and context (SC vs. PP vs. FA) from non-parametric, Kruskall-Wallis test. A *p*-value > 0.05 indicates no difference in scanner trueness according to the context

**Fig. 7:**
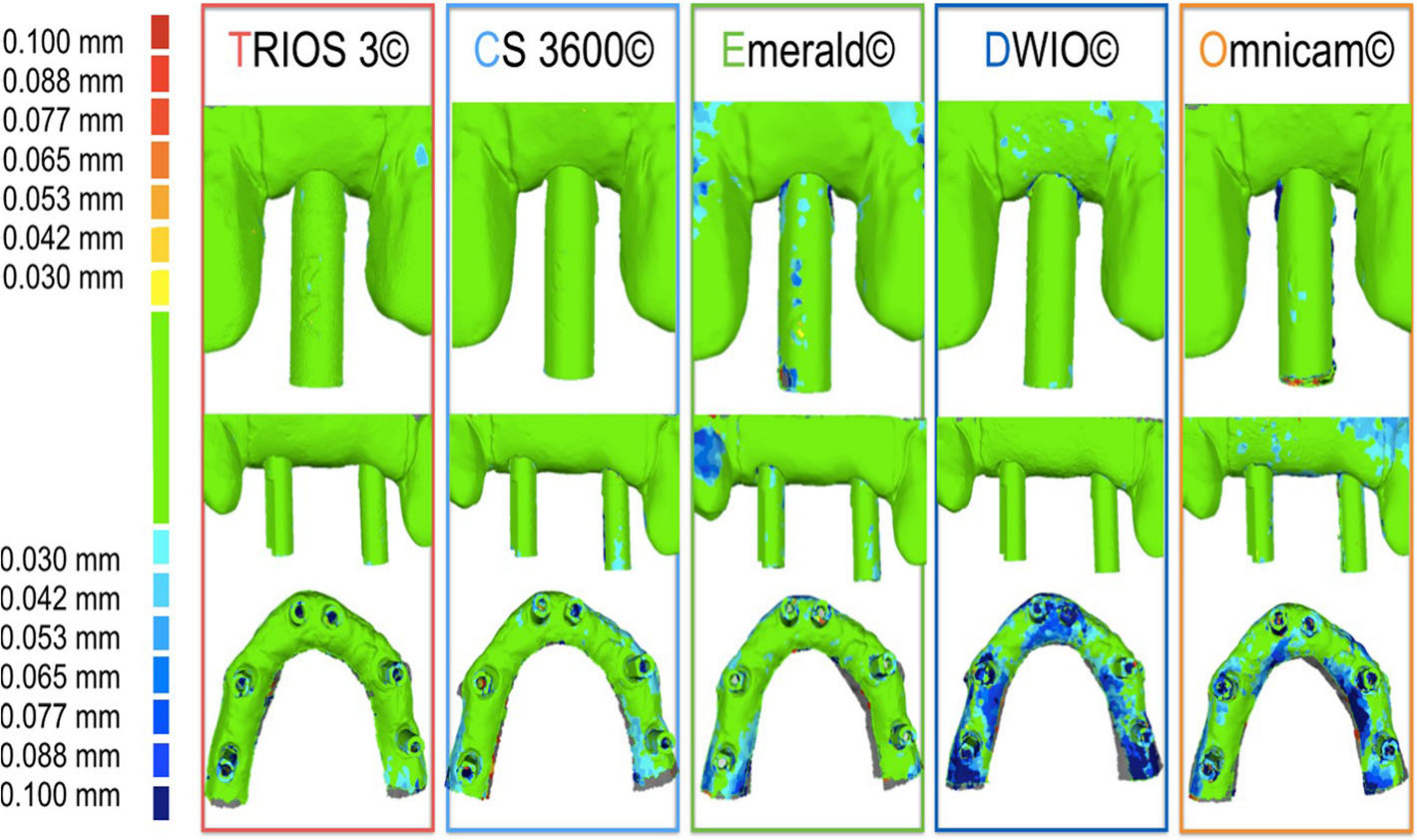
Precision in the single crown (SC), partial prosthesis (PP) and full-arch (FA) with the 5 examined intraoral scanners (IOs): colorimetric maps. The color maps indicated inward (blue) or outward (red) displacement between overlaid structures, whereas a minimal change was indicated by a green color. For all three models (SC, PP, FA): the color scale ranged from a maximum deviation of + 100 μm and − 100 μm, with the best result given by the deviations comprised between + 30 μm and − 30 μm (green color)

**Fig. 8:**
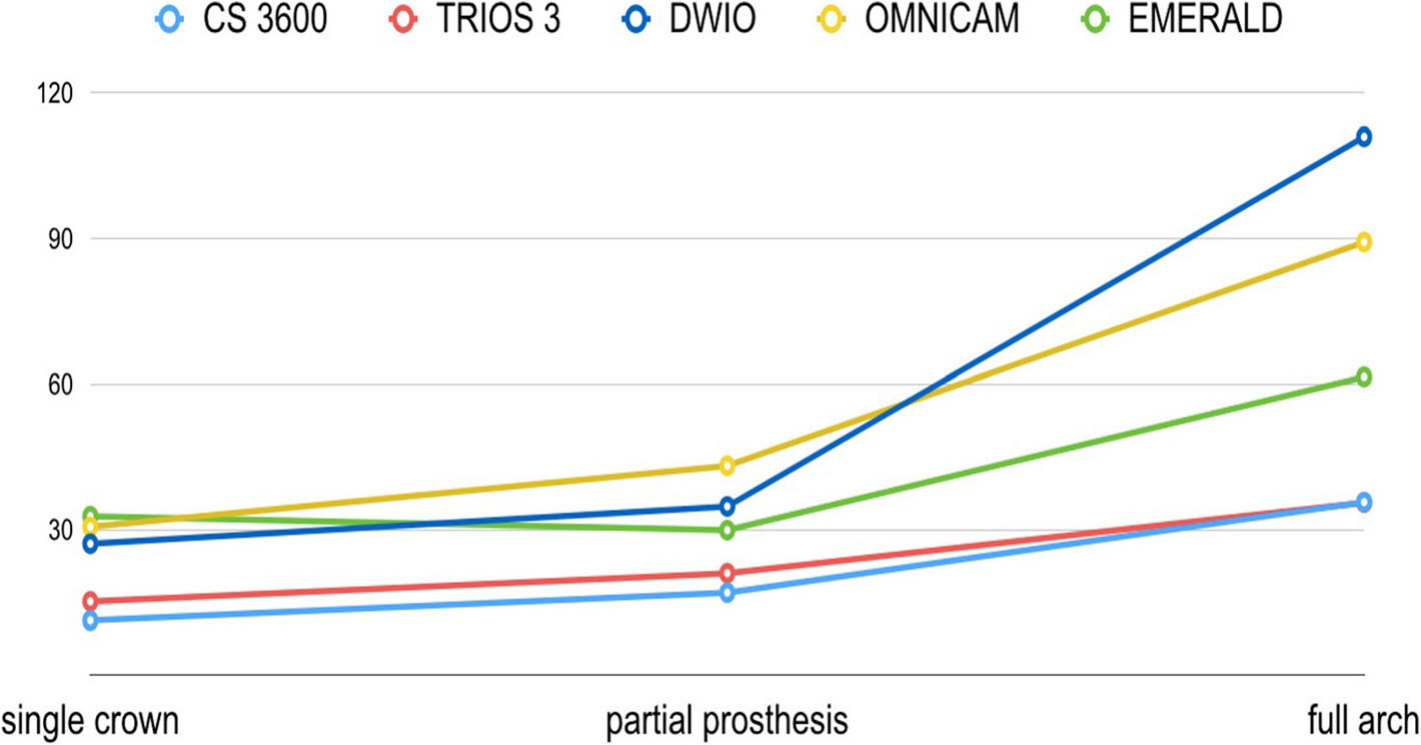
Changes in mean precision (standard deviation), in μm, for the 5 examined scanners, in the different applications (single crown, SC vs. partial prosthesis, PP vs. full-arch, FA)

The precision results are summarized in Tab. 3 and in Figs. 7 and 8. In brief, in the SC, CS 3600® had the best precision (11.3 ± 1.1 μm), followed by Trios3® (15.2 ± 0.8 μm), DWIO® (27.1 ± 10.7 μm), Omnicam® (30.6 ± 3.3 μm), and Emerald® (32.8 ± 10.7 μm). CS 3600® and Trios3® were statistically more precise than DWIO®, Omnicam®, and Emerald®. In the PP, CS 3600® had the best precision (17 ± 2.3 μm), followed by Trios3® (21 ± 1.9 μm), Emerald® (29.9 ± 8.9 μm), DWIO® (34.8 ± 10.8 μm), and Omnicam® (43.2 ± 9.4 μm). CS 3600® was statistically more precise than Emerald®, DWIO®, and Omnicam®; while Trios3® was statistically more precise than DWIO and Omnicam; and Emerald was statistically more precise than Omnicam®. Finally, in the FA, Trios3® had the best precision (35.6 ± 3.4 μm), followed by CS 3600® (35.7 ± 4.3 μm), Emerald® (61.5 ± 18.1 μm), Omnicam® (89.3 ± 14 μm), and DWIO® (111 ± 24.8 μm). CS 3600® and Trios3® were statistically more precise than Emerald®, Omnicam®, and DWIO®; while Emerald® was statistically more precise than Omnicam® and DWIO®; and Omnicam® was statistically more precise than DWIO®. A statistically significant different in precision was found, for each scanner, between the different contexts (SC vs. PP vs. FA).

**Table 3:** Mean precision and its standard deviation (SD) in micrometers (μm) with single crown (SC), partial prosthesis (PP) and full-arch (FA), and *p* values testing the scanner by context interaction. *N* = 10 scans for each scanner and implant type

Scanner	Single Crown (SC)	Partial prosthesis (PP)	Full arch (FA)	*p*-value^1^
	Mean ± SD	Mean ± SD	Mean ± SD	
Trios 3®	15.2 ± 0.8†,‡,•	21.0 ± 1.9‡,•	35.6 ± 3.4†,‡,•	<.0001
CS 3600®	11.3 ± 1.1^,§,#	17.0 ± 2.3^,§,#	35.7 ± 4.3^,§,#	<.0001
Emerald®	32.8 ± 10.7†,^	29.9 ± 8.9^,°	61.5 ± 18.1†,^,°,*	0.0007
DWIO®	27.1 ± 10.7‡,§	34.8 ± 10.8‡,§	111.0 ± 24.8‡,§,°,^ç^	<.0001
Omnicam®	30.6 ± 3.3•,#	43.2 ± 9.4•,#,°	89.3 ± 14.0•,#,*,^ç^	<.0001

The same symbol after SD indicates differences in precision between scanner pairs (Tukey-adjustment for multiple comparison). Minimum significant difference across scanners: 8.8 μm, 9.8 μm, 19.4 μm for single crown (SC), partial prosthesis (PP) and full arch (FA), respectively. ^1^*p*-value testing the interaction between scanner and context (SC vs. PP vs. FA) from non-parametric, Kruskall-Wallis test. A *p*-value > 0.05 indicates no difference in scanner precision according to the context

## Discussion

To date, only a few studies have compared the accuracy of different IOSs in implantology [9–11, 26–28].

Van der Meer and colleagues compared three different IOSs (CEREC AC Bluecam®, iTero®, and Lava COS®) in a partially edentulous model with 3 implants [27]. The implants were connected with PEEK scanbodies, 10 scans were taken for each IOS, and all of these were loaded into reverse-engineering software, where the distances and angles between the different cylinders were calculated [27]. These values were compared with reference measurements obtained with an industrial 3D scanner. Considering the linear distances, Lava COS® showed the minor deviations, CEREC® the major [27]. Angular deviations were minimal in all IOSs [27]. The authors concluded that an increase in linear and angular errors is to be expected with all IOSs, over the length of the arch as well as on the accumulation of patched 3D surfaces [27].

In another in vitro study, two representative models of a PEM and TEM were prepared, with three and six PEEK scanbodies, respectively [10]. These models were scanned with four different IOSs (Trios2®, CS 3500®, Zfx Intrascan®, and Planscan®), five scans for each of the scanners; the models were then superimposed via reverse-engineering software to the RMs, captured with a powerful industrial scanner, in order to evaluate the general trueness [10]. In addition, the distance and angles between simulated implants were measured in each group and compared to those of the RM, to evaluate local trueness [10]. Finally, the precision was calculated by overlapping the scans captured with the different IOSs, within each group. General trueness and precision of any IOSs were compared by model type, through an ANOVA model including scanner, model, and their interaction [10]. At the end of the study, CS 3500® had the best general trueness (47.8 μm) and precision (40.8 μm) in the PEM, followed by Trios2® (trueness 71.2 μm; precision 51.0 μm), Zfx Intrascan® (trueness 117.0 μm; precision 126.2 μm), and Planscan® (trueness 233.4 μm; precision 219.8 μm) [10]. The study highlighted statistically significant differences between the different IOSs in the PEM, as well as in the TEM [10]. In the TEM, CS 3500® had the best performance in terms of general trueness (63.2 μm) and precision (55.2 μm), followed by Trios2® (trueness 71.6 μm; precision 67.0 μm), Zfx Intrascan® (trueness 103.0 μm; precision 112.4 μm), and Planscan® (trueness 253.4 μm; precision 204.2 μm) [10].

More recently, Imburgia and colleagues have published another in vitro study with a similar structure and setting [9], comparing four different and modern IOSs (CS 3600®, Trios3®, Omnicam®, and TrueDefinition®). The authors prepared models with (respectively) three (partially edentulous model, PEM) and six implant analogs (totally edentulous model, TEM), on which PEEK scanbodies were screwed. Once again, the models were scanned with an industrial scanner to obtain. STL files of reference, onto which the individual intraoral scans captured with the different IOSs were superimposed, in order to evaluate trueness [9]; finally, the IOS models were superimposed on each other within groups, to determine precision. At the end of the study, CS3600® had the best trueness (45.8 ± 1.6 μm) in the PEM, followed by Trios3® (50.2 ± 2.5 μm), Omnicam® (58.8 ± 1.6 μm), and TrueDefinition® (61.4 ± 3.0 μm) [9]. In the TEM, CS 3600® had the best trueness (60.6 ± 11.7 μm), followed by Omnicam® (66.4 ± 3.9 μm), Trios3® (67.2 ± 6.9 μm), and TrueDefinition® (106.4 ± 23.1 μm) [9]. With regard to precision, TrueDefinition® had the best precision (19.5 ± 3.1 μm) in the PEM, followed by Trios3® (24.5 ± 3.7 μm), CS 3600® (24.8 ± 4.6 μm), and Omnicam® (26.3 ± 1.5 μm); conversely, in the TEM, Trios3® had the best precision (31.5 ± 9.8 μm), followed by Omnicam® (57.2 ± 9.1 μm), CS 3600® (65.5 ± 16.7 μm), and TrueDefinition® (75.3 ± 43.8 μm) [9]. The study revealed statistically significant differences between the various IOSs examined, both in terms of trueness and precision; moreover, differences were found among the different applications, with the best results obtained for the PEM when compared to the TEM. This confirms the evidence emerging from previous studies in the literature [11, 26–28] that have shown how the error in the intraoral scan increases progressively with the increase of the scanned area.

In our present in vitro study, which represents the evolution of the aforementioned studies [9, 10], all IOs showed high trueness, and a rather small deviation from the RM, in the single implant scan. In fact, four out of five scanners (CS 3600®, Trios3®, DWIO®, and Omnicam®) showed an error below the critical threshold, set at 30 μm. In particular, CS 3600® had a mean error of 15.2 μm (±0.8), followed by Trios3® (22.3 ± 0.5 μm), DWIO® (27.8 ± 3.2 μm), and Omnicam® (28.4 ± 4.5 μm). Furthermore, the SDs or variations within each of the groups were very small, confirming a high reliability and repeatability of results, in the single implant scan. In this specific application, only the Emerald® scanner had a mean error of more than 30 μm, with an average truth value of 43.1 μm and a rather high SD (11.5). However, this error is in any case compatible with the design (and thus the manufacture and clinical application) of an implant-supported SC. In any case, already from the SC, statistically significant differences were found between the different scanners. In particular CS 3600® was statistically truer than DWIO®, Omnicam®, and Emerald®; moreover Trios3®, DWIO®, and Omnicam® were statistically truer than Emerald. The primacy of CS 3600® and Trios3® was also confirmed by the results obtained in the scan on two implants, for the design of a bridge of three elements (PP). In fact, in trueness, CS 3600® had a mean error of 23.0 μm (±1.1), with Trios3® showing a slightly higher error (28.5 ± 0.5 μm). The stability of the result within the 10 measurements for each of these two scanners was remarkable; both, among other things, presented for this specific application an error lower than the critical threshold of 30 μm. Omnicam® followed, with an error of 38.1 μm (±8.8), while Emerald® (49.3 ± 5.5 μm) and DWIO® (49.8 ± 5.0 μm), practically paired, were more distant. From the statistical point of view, once again, there were clear differences between the scanners analyzed. In particular, CS 3600® and Trios3® were statistically truer than Omnicam®, Emerald®, and DWIO®; moreover, Omnicam® was statistically truer than Emerald® and DWIO®. Globally, in any case, these results were, for all the scanners, compatible at least in theory (and without prejudice to the subsequent error in the CAM phase) with the fabrication of a bridge of three elements. It was rather interesting to evaluate how, in all the IOSs, the error grew with the passage from a single implant scan to a scan of two implants. The average error growth was 6.2 μm (Trios 3® and Emerald®), 7.8 μm (CS 3600®), 9.7 μm (Omnicam®), and 22 μm (DWIO®), respectively. Evidently, all the IOSs showed a good stability of result, in terms of trueness, in the transition from a single implant scan to a scan of two implants; the only scanner that seemed to present more difficulties in this sense was DWIO, with a greater gap than all the others. From the statistical point of view, anyway, there was a significant difference between a single implant and two implants, for all the scanners. Finally, in the scan of six implants for the design and manufacture of a fixed FA prosthesis, the best result in trueness was that of the CS 3600® (44.9 ± 8.9 μm), which was confirmed as the best scanner for this application, followed very closely by Trios3® (46.3 ± 4.9 μm). Surprising, then (although detached from the first two), was the result of Emerald®, with a trueness in the acquisition of six implants in the completely edentulous patient of 66.3 μm (±5.6). Omnicam® (70.4 ± 11.8 μm) and DWIO® (92.1 ± 24.1 μm) followed that; due to the greater error and the poor repeatability of results, these two scanners appeared the most difficult to use for the manufacture of a FA prosthesis. In light of all this, from a statistical point of view, CS 3600® and Trios3® were statistically truer than Emerald®, Omnicam®, and DWIO®; while Emerald® and Omnicam® were statistically truer than DWIO®. Once again, it was also interesting to evaluate the difference between the scan on two implants (for the design of a three-unit bridge) and the scan on six implants (for the design of a FA fixed prosthesis). In this sense, the average error in all IOSs increased (respectively) by 17 μm (Emerald®), 17.8 μm (Trios3®), 21.9 μm (CS 3600®), 32.3 μm (Omnicam®), and 42.3 μm (DWIO®). With regard to this, the best result was achieved by Emerald®, which confirmed a pattern of high stability in the comparison between quality of different scans (single implant vs. two implants vs. six implants), closely followed by Trios3®. In any event, there was a significant difference between two and six implants, for all the scanners.

What, then, are the main evidences that emerge from this study, at the level of trueness? First of all is the exceptional performance of all IOSs investigated in scanning for SCs and short-span restorations on implants. The results obtained in the present study are in fact fully compatible with the realization, through a careful digital workflow in the subsequent CAD and CAM phases, of high-quality restorations with satisfactory marginal gaps. Only in the TEM model did the results seem not yet fully compatible with the realization of a FA, as also reported in the literature [20, 21]. However, if we compare the trueness of CS 3600® and Trios3® in the FA, in the present study, with the results obtained in the previous work of Imburgia and colleagues [9], we note how the improvements introduced by the new versions of the acquisition software of these scanners are substantial: the error is reduced from 60 μm to 44 μm for CS 3600® and from 67 μm to 46 μm for Trios3®. Conversely, from the comparative analysis of the results obtained in the present study with those reported by Imburgia and colleagues [9], it emerges that the results obtained by Omnicam are stable; this is obvious since the version of the acquisition software used is identical in the two studies. Planmeca, instead, made a decisive leap forward with the new hardware (Emerald®) compared to the previous scanner (Planscan®). Finally, a last interesting element that emerges from the present study is how the accuracy does not seem to be related in any way to the resolution of acquisition. In fact, the CS 3600® was the most accurate scanner, but also the one with the lowest acquisition resolution (fewer triangles making up the meshes, in all applications). In implantology the number of triangles that make up the mesh seems to be of lesser importance than accuracy: the optical impression aims to capture a position [13]. With natural teeth is different: in that context, a higher resolution of acquisition contributes to making visible the margin of the prosthetic preparation [12].

From the point of view of precision, the results were excellent for all IOSs, at least for SC and PP, with minimal errors, and were contained within the 30-μm range. Only Omnicam® (30.6 ± 3.3 μm) and Emerald® (32.8 ± 10.7 μm) showed deviations slightly higher than 30 μm in the SC; in the PP, they were DWIO® (34.8 ± 10.8 μm) and Omnicam® (43.2 ± 9.4 μm) to deviate beyond the 30-μm threshold. Deviations grew, of course, in the FA, where all the IOSs showed errors of more than 30 μm. These errors were contained for Trios3® (35.6 ± 3.4 μm) and CS 3600® (35.7 ± 4.3 μm), more marked for Emerald® (61.5 ± 18.1 μm), Omnicam® (89.3 ± 14 μm), and DWIO® (111 ± 24.8 μm). Even in precision, statistically significant differences emerged between the different machines examined.

Our study has limits. First of all, it is an in vitro study. Although it is not possible, to date, to determine the trueness and therefore the accuracy of an IOS in vivo, it should not be forgotten that there are important factors that can differentiate the quality of a scan on a plaster model from that of a scan in the patient’s mouth. Variations in measurements between in vitro and in vivo may be important and depend not only on the presence of blood and saliva, but above all on the technical difficulty of the intraoral acquisition, as well as on the patient’s movements and the peculiar optical behavior of dental tissues [30–32]. The teeth, being made of enamel and dentin, have a different optical behavior from that of gypsum models; this does not help the IOS in reading and rebuilding the mesh. In a recent study, Albdour et al. [33] cautioned that the trueness of the IOS in vivo may be less than that shown in vitro (on plaster models). Although these considerations are probably of greater importance when capturing the impression on the natural tooth (with implants we mainly capture the position of scanbodies, made of PEEK), we must not forget that the presence of adequate contact points is key in prosthetic rehabilitation with implant-supported SCs or fixed PP. Another limitation of the present study is our having used an optical desktop scanner as a tool for capturing RMs. This desktop scanner, although of an industrial derivation and with a certified accuracy of 5 μm, does not have the same accuracy as a probe. Furthermore, another limit of the present study could be the scanning strategy. The scanning method used (zig-zag) could be more suitable for some of the IOSs analyzed in this study, while penalizing others; however, since neither the literature [11, 34] nor the companies themselves provide details on the ideal scanning strategy, in this paper we have extended the same protocol to all IOSs analyzed. Finally, an inherent limitation of all comparative studies on IOSs is the fact that a new acquisition software release is sufficient to improve (or worsen) the accuracy of a machine considerably. As companies continue to improve their products and release new software, it is possible that our current study may not reflect the accuracy of the most up-to-date machines currently on the market. To overcome this problem, however, we have specified in the text (under Methods) the version of the acquisition software used for each scanner. Moreover, in our present work, only 5 IOSs have been evaluated, while new machines are introduced on the market every month, with more than 20 scanners already available today. Ideally, a comprehensive study should include as many IOSs already on the market as possible. However, for reasons of time, and given the great amount of data to be processed, in this work we limited ourselves to 5 IOSs that we considered modern, deliberately excluding the older devices that used powder to capture the mesh. This was a precise choice, due to the fact that powder represents a major limitation in terms of accuracy and clinical use [35]; nevertheless, we are aware of the fact that new machines recently introduced on the market—for example the Primescan® from Dentsply-Sirona, the Trios4® from 3-Shape, the CS 3700® from Carestream, the Virtuo-Vivo® from Dentalwings or the Korean scanner Medit i500®—must necessarily be studied, in order to understand the real mathematical reliability and whether they can ensure further technological advancement to digital dentistry. The analysis of the new machines introduced to the market can and should be the subject of the next comparative studies of IOSs.

## Conclusions

Since only a few studies have compared the accuracy of different IOSs in implantology, the aim of our present in vitro work was to compare the trueness and precision of 5 different scanners in the impressions of single and multiple implants. Hence, two plaster models were prepared, representative of three clinical situations: a single crown (SC), a partial prosthesis (PP), and a full-arch (FA). These models were scanned with a desktop scanner, to capture reference models (RMs), and then with different 5 IOSs (CS 3600®, Trios3®, Omnicam®, DWIO®, Emerald®); 10 scans were taken for each model, using each IOS. All IOS datasets were loaded into reverse-engineering software where they were superimposed on the corresponding RMs, to evaluate trueness, and superimposed on each other within groups, to determine precision. At the end of the study, the five IOSs examined showed significant differences between them; in addition, the mathematical error increased in the transition from SC to PP up to FA. Both these data seem to confirm what reported in the literature, and this has relevant clinical implications because from this study we can draw indications for the use of different IOSs, in different clinical contexts. However, we must not forget that this is an in vitro study, and the evidence emerging from this work must be confirmed in the clinics.

## Data Availability

The .STL files and the 3D surface models obtained in this study with the different five IOS as well as the reference files obtained with the desktop scanner belong to the authors, and are therefore available only upon reasonable request, after approval by all the authors.

## Abbreviations

CAD: Computer-assisted-design
CAM: Computer-assisted-manufacturing
CBCT: Cone beam computer tomography
CMM: Coordinate measuring machine
FA: Full-arch
IOS: Intraoral scanner
PEEK: Polyether-ether-ketone
PEM: Partially edentulous model
PP: Partial prosthesis
RICP: Robust-iterative-closest-point
RM: Reference model
SC: Single crown
SD: Standard deviation
SSS: Stable scan stage
STL: Standard triangulation language
TEM: Totally edentulous model
